# Facial and Thoracic Deformities Incident to Obstruction by Adenoid Hypertrophy in the Naso-Pharynx

**Published:** 1890-11

**Authors:** W. E. Casselberry

**Affiliations:** Chicago


### SELECTIONS.

### Facial and Thoracic Deformities Incident to Obstruction by Adenoid Hypertrophy in the Naso-Pharynx.

BY W. E. CASSELBERRY, M.D., CHICAGO.

Extracts from a paper read before the American Medical Association meeting of 1890.

" The space of the naso-pharynx is, by nature's law, designed to be free, and to serve as a common area of air communication between the five openings which enter it."

" Into this space also open the posterior nares, the natural respiratory passage between *via* the nose and naso-pharynx. Adenoid hypertrophy, therefore, serves as a plug to the posterior nasal openings, and obstructs nasal respiration completely or in part according to the degree of glandular enlargement. From this point we find it a matter of exceeding interest to trace the origin and development of each successive step in the series of deformities consequent upon this condition. The plugging up of the posterior nares necessitates oral breathing, and the constantly open mouth interferes with the normal adaptation of certain facial muscles, which in turn effects radical changes in the contour of the soft and developing bones of the face, the whole resulting in a physiognomy characterized by a vacant, stupid, almost idiotic expression of countenance."

" The hanging lower jaw causes the face to appear elongated. The nose is pinched, or its alae distended, while the angles of the mouth and eyes have a drawn appearance."

" Moreover, as pointed out by Henri Chatellier, of Paris, cited by Hooper, of Boston, the air cavities in communication with the nose as the frontal, maxillary, sphenoidal, and ethmoidal sinuses, which are essential to the proper expansion of their respective bones, cease to develop when the circulation of air through the nose is interfered with, thus altering nature's intent regarding the dimensions of the face and head, and still further deforming the physiognomy. Dr. Hooper has also described and illustrated the next link in the chain of deformities—the high-arched hard palate and V-shaped indenture. The naturally rounded arch of the roof of the mouth is formed, in large part, by the palate process of the superior maxillary bone, which also constitutes a corresponding portion of the floor of the nose. Augmentation of atmospheric pressure upon the buccal surface of the palate process and the impact of air currents to and fro during mouth-breathing, together with diminution of intra-nasal air pressure incident to nasal obstruction, gradually forced upward, the centre of the hard palate, and change thus, the obtusely rounded Romanesque arch into one of Gothic shape—the pointed or high-arched palate invariably existing in association with long continued and excessive adenoid development during childhood. Elevation of the palatal arch lessens the transverse diameter of the jaw and causes it to grow pointed in front—the so-called V-shaped indenture—and with the resulting contraction of the alveolar process, the teeth, especially those near the point, are crowded into various grotesque aggregations, or are rotated on their axes."

"Dentists have long recognized what they call the "habit" of mouth-breathing as a prolific source of irregular indentures."

"We would advance a step and assert that mouth-breathing is but rarely a "habit." It conduces to so much discomfort that even the babe would feel inclined to close this aperture could he secure the requisite amount of air through the nose and naso-pharynx. Aside from greatly debilitated states, it is occlusion to some degree of these passages which necessitates the habit."

"Next, elevation of the palatal arch must produce contortion within the nose. Deflections of the septum are rarely congenital."

"Designed by nature to fill vertically the natural space between the roof of the nose and its floor, the abbreviation of this space by elevation of the palatal arch through the instrumentality of naso-pharyngeal adenoid hypertrophy can not result otherwise than in forcing the septum to provide for itself by bending and curving laterally in various directions."

"Furthermore, guided by the contorted septum, even the external nose may be twisted to one side— a deformity common enough in its milder aspects, and not unfrequently seen in aggravated forms."

"Finally, not only, as before said, do these unfortunates *look* stupid, but they really *are* stupid and exhibit abundant evidence of mental hebetude, with inability to fix the attention to learn, to memorize, or to reason, the whole evidencing an impairment of cerebral function. Indeed, we hold it not illogical to believe that in extreme cases of long duration, associated perhaps with deafness, such alteration of cerebral function might ensue as to result in absolute idiocy."

